# Inflammatory Reactions Within the Epicardial Adipose Tissue Are Associated with the Expression of the Receptor for Advanced Glycation End Products in Aortic Stenosis

**DOI:** 10.3390/jcm15020428

**Published:** 2026-01-06

**Authors:** Atsunobu Oryoji, Kosuke Saku, Nobuhiro Tahara, Sho-ichi Yamagishi, Eiki Tayama

**Affiliations:** 1 Division of Cardiovascular Surgery, Department of Surgery, Kurume University School of Medicine, Kurume 830-0011, Japan; ouryouji_atsunobu@kurume-u.ac.jp (A.O.); eiki@kurume-u.ac.jp (E.T.); 2 Division of Cardiovascular Medicine, Department of Medicine, Kurume University School of Medicine, Kurume 830-0011, Japan; 3 Division of Diabetes, Metabolism, and Endocrinology, Department of Medicine, Showa Medical University School of Medicine, Tokyo 142-8666, Japan; shoichi@med.showa-u.ac.jp

**Keywords:** aortic stenosis, epicardial adipose tissue, receptor for advanced glycation end products, inflammation, macrophage

## Abstract

**Background:** Epicardial adipose tissue (EAT) is a metabolically active organ implicated in coronary artery disease (CAD); however, its role in aortic stenosis (AS) remains unclear. Advanced glycation end products (AGEs) and their receptor (RAGE) promote cardiovascular tissue inflammation. This study aimed to investigate whether inflammatory activity within the EAT, particularly involving the AGEs-RAGE axis, is associated with AS. **Methods:** We studied 42 patients (isolated AS, *n* = 15; AS with CAD, *n* = 15; and CAD alone, *n* = 12) undergoing surgical intervention, along with 10 autopsy controls. EAT volume was assessed via computed tomography and indexed to body surface area. Furthermore, macrophage infiltration (CD68) and RAGE expression in EAT samples were analyzed using immunohistochemistry and immunofluorescence imaging. **Results:** EAT volume index was significantly higher in all surgical groups than in the controls (*p* < 0.001). These surgical groups also had markedly increased CD68- and RAGE-positive cells compared with the controls (*p* < 0.001), with colocalization detected by means of immunofluorescence imaging. Additionally, the EAT volume index independently and positively correlated with CD68-positive cell counts (*p* = 0.021), and causal mediation analysis suggested that it promotes CD68-positive macrophage activation through pathways mediated by RAGE-positive cells (*p* = 0.024). Inflammatory cells did not correlate with AS severity (maximum aortic jet velocity, mean pressure gradient, aortic valve area). **Conclusions:** EAT in AS exhibits increased macrophage infiltration and RAGE expression. Therefore, the AGEs-RAGE axis may contribute to local inflammatory activity, and EAT can be a potential biomarker and therapeutic target in AS.

## 1. Introduction

Calcified aortic stenosis (AS) is the most common acquired valvular disorder in developed countries, with its prevalence reaching 50% in individuals aged over 85 years [1,2]. Historically considered as a passive degenerative process, AS progression reportedly involves active mechanisms, including fibrogenesis and osteogenesis within the aortic valves [3]. Indeed, cytokines such as interferons and tumor necrosis factor (TNF)-α have been linked to inflammation-driven valvular calcification [3]. Under inflammatory and oxidative stress conditions, valvular interstitial cells differentiate into myofibroblasts and osteoblast-like cells, resembling atherosclerosis-related processes [4].

Advanced glycation end products (AGEs) are nonenzymatic macromolecular derivatives formed via the Maillard reaction [5,6]. A receptor for AGEs (RAGE), an immunoglobulin superfamily member, mediates the pathological signaling pathways of AGEs [5,6]. When activated by AGEs, RAGE drives oxidative stress, triggering inflammatory and thrombogenic responses in endothelial cells, smooth muscle cells, and macrophages [5,6]. Consequently, the AGEs-RAGE axis has been linked to various pathologies, including diabetic vascular complications and cardiovascular disease, Alzheimer’s disease, cancer, insulin resistance, and nonalcoholic fatty liver disease [5,6].

Epicardial adipose tissue (EAT) is an endocrine and paracrine organ that modulates inflammatory and atherogenic pathways by secreting adipokines, thereby contributing to coronary artery disease (CAD) pathogenesis [7,8,9]. Notably, patients with AS demonstrate increased EAT volume compared with non-AS controls, independent of age, sex, or cardiovascular risk factors [10]. However, the pathophysiological relationship between EAT and AS remains unclear. Thus, this study aimed to investigate whether inflammatory reactions evaluated by CD68, a marker of macrophages, and RAGE expression within the EAT are associated with AS.

## 2. Materials and Methods

### 2.1. Study Population

We enrolled patients with moderate and severe AS undergoing surgical aortic valve replacement (SAVR) (isolated AS group), patients with concomitant AS and CAD undergoing SAVR plus coronary artery bypass grafting (CABG) (AS+CAD group), and patients with CAD undergoing CABG alone (CAD group) at Kurume University Hospital between March 2020 and July 2023. Autopsied individuals who had died from malignant or hematologic diseases and had undergone autopsy based on consent served as controls. The absence of cardiovascular disease, including AS, was confirmed by medical history, electrocardiography, and transthoracic echocardiography performed before death. Conversely, we excluded patients with Marfan syndrome, other connective tissue disorders, acute or chronic aortic dissection, aortitis, and re-do SAVR cases. AS severity was based on the American Heart Association/American College of Cardiology guidelines [11]. The Ethical Committee for Clinical Research of Kurume University (No. 19210) approved this study, and all patients provided written informed consent.

### 2.2. Clinical Variables

Demographic data and information on medical history, medication use, and smoking status were obtained using a questionnaire. Blood pressure and resting heart rate were measured using standard methods after participants avoided vigorous activities and smoking for ≥60 min. To measure the ankle-brachial index, we used an automated device (VP-1000; Omron Healthcare Co., Ltd., Kyoto, Japan) [12]. Echocardiography was performed according to the American Society of Echocardiography guidelines, with peak velocity, mean pressure gradient, velocity time integral, and aortic valve area calculated using the continuity equation. For assessing left ventricular ejection fraction, the modified biplane Simpson method was employed. Blood samples were drawn from the antecubital vein preoperatively.

### 2.3. EAT Assessment

**Figure 1:**
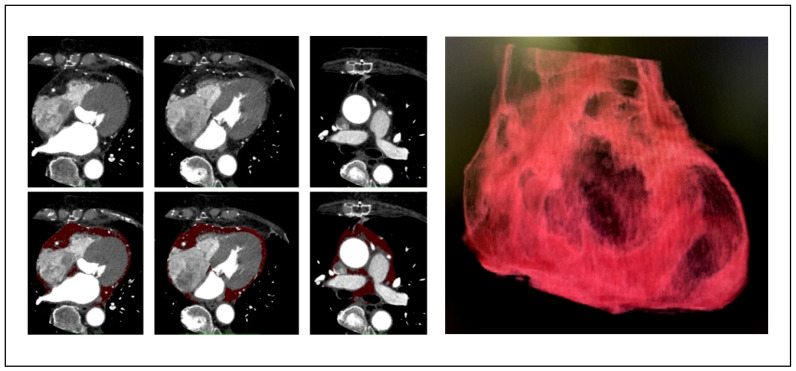
Definition of EAT on CT. EAT was defined as adipose tissue between the myocardium and pericardium. Representative axial CT images show EAT (highlighted area) with attenuation values of −195 to −15 Hounsfield units. EAT, epicardial adipose tissue; CT, computed tomography.

The adipose tissues between the myocardium and pericardium on 64-slice computed tomography (CT) (Discovery CT750HD, GE HealthCare, Chicago, IL, USA) defined the EAT. Figure 1 presents representative axial CT images showing EAT tracing. For volumetric analysis, CT datasets were transferred to a dedicated workstation (Ziostation 2, Ziosoft, Tokyo, Japan). The pericardial contour was semi-automatically traced on each axial slice from the level of the main pulmonary artery bifurcation to the diaphragm. Adipose tissue voxels within the pericardial sac were identified using a predefined attenuation threshold ranging from −195 to −15 Hounsfield units, as previously validated for EAT quantification [10]. The EAT volume index (mL/m^2^) was defined as EAT volume (mL)/body surface area (m^2^).

### 2.4. Histopathology

EAT specimens were collected intraoperatively from patients with AS, CAD, and AS+CAD, and from autopsied individuals who served as controls, for analyses of CD68 and RAGE expression. Immunohistochemical and immunofluorescence staining methods were applied as previously described [13]. Briefly, EAT specimens were fixed in neutral buffered formalin and then embedded in paraffin sections. For immunohistochemical staining, tissue sections were sliced 4-μm thick. RAGE and CD68 in EAT were evaluated with immunohistochemical or immunofluorescent staining. Primary antibodies such as anti-RAGE (SC-365154, Santa Cruz Biotechnology, Santa Cruz, CA, USA) and anti-CD68 (IS613, Dako/Agilent Technologies, Inc., Santa Clara, CA, USA) were used in the experiments. The nucleus was stained using Mayer’s hematoxylin and a fluorescent dye, TO-PRO3 (T3605, Thermo Fisher Scientific, Waltham, MA, USA). Digital images were obtained using a microscope attached to imaging software (KEYENCE BZ-9000, KEYENCE, Osaka, Japan). Positive cells per mm^2^ were quantified using five digital images per site (1360 × 1024 pixels, 362 × 273 µm) [14].

### 2.5. Statistical Analysis

Data are presented as mean ± standard deviation or median (interquartile range). Data normality was assessed using the Shapiro–Wilk test. Parametric or nonparametric tests were applied as appropriate. Categorical variables were compared using χ^2^ tests, whereas continuous variables were compared among groups using analysis of variance with Tukey’s post hoc test. We used log-transformed values for skewed data.

Correlations were evaluated via univariable and multivariable regression analyses. Clinically relevant variables were included in the multivariable model. A *p* value below 0.05 indicated statistical significance. We also conducted a causal mediation analysis using a multivariable regression–based approach to test whether increased EAT volume index promotes CD68-positive macrophage activation via RAGE. In this analysis, the exposure and outcome variables were the EAT volume index and CD68-positive cell counts, respectively, with the number of RAGE-positive cells as the putative mediator. All statistical data were analyzed using JMP 18 (SAS Institute, Cary, NC, USA).

## 3. Results

### 3.1. Patient Characteristics

This study included 42 patients (15 isolated AS, 15 AS+CAD, 12 CAD) and 10 controls. The isolated AS and AS+CAD groups were older than the other groups without AS, with more females than males. While hypertension and dyslipidemia were similarly prevalent across the groups, diabetes mellitus was more common in the CAD group. Lipid profiles and left ventricular size and function were comparable among the surgical groups. Both the AS and AS+CAD groups exhibited left ventricular hypertrophy (Table 1).

### 3.2. EAT Volume

**Figure 2:**
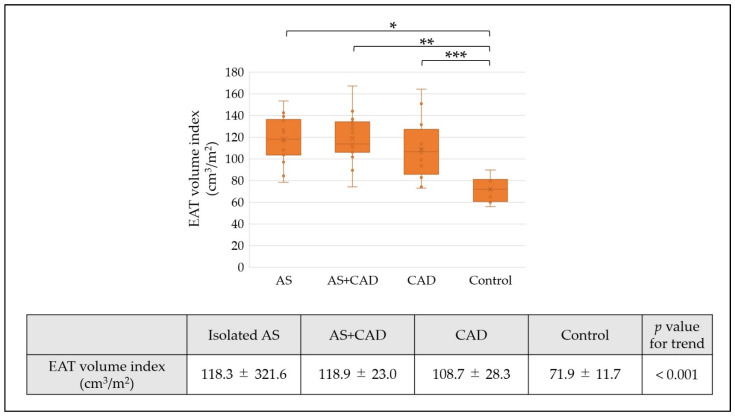
EAT volume index among study groups. EAT volume index in isolated AS, AS+CAD, CAD, and control groups. Data are presented as mean ± standard deviation. * AS vs. control: *p* < 0.001, ** AS+CAD vs. control: *p* < 0.001, *** CAD vs. control: *p* = 0.003. EAT, epicardial adipose tissue; AS, aortic stenosis; CAD, coronary artery disease.

The EAT volume was similar across surgical groups (isolated AS, 174.7 ± 32.8 cm^3^; AS+CAD, 182.3 ± 38.4 cm^3^; and CAD, 183.7 ± 42.6 cm^3^) and significantly higher than that in controls (112.2 ± 17.1 cm^3^, each surgical group vs. control: *p* < 0.001). The EAT volume index demonstrated a similar trend across the surgical groups: isolated AS, 118.3 ± 321.6 cm^3^/m^2^; AS+CAD, 118.9 ± 23.0 cm^3^/m^2^; CAD, 108.7 ± 28.3 cm^3^/m^2^; and control, 71.9 ± 11.7 cm^3^/m^2^ (Figure 2, AS vs. control: *p* < 0.001, AS+CAD vs. control: *p* < 0.001, CAD vs. control: *p* = 0.003).

### 3.3. CD68 and RAGE Expression

**Figure 3:**
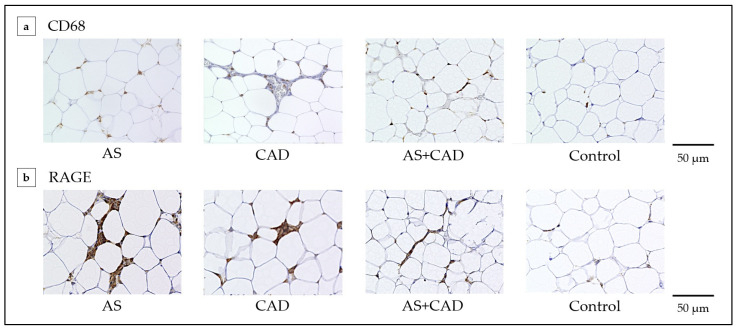
Immunohistochemical staining for CD68 and RAGE in EAT. (**a**) Representative immunohistochemical images of CD68 expression in EAT from the isolated AS, CAD, AS+CAD, and control groups. (**b**) Representative immunohistochemical images of RAGE expression in EAT from the isolated AS, CAD, AS+CAD, and control groups. CD68-positive and RAGE-positive cells are localized around adipocyte nuclei. Scale bars: 50 µm. EAT, epicardial adipose tissue; AS, aortic stenosis; CAD, coronary artery disease; RAGE, receptor for advanced glycation end products.

**Figure 4:**
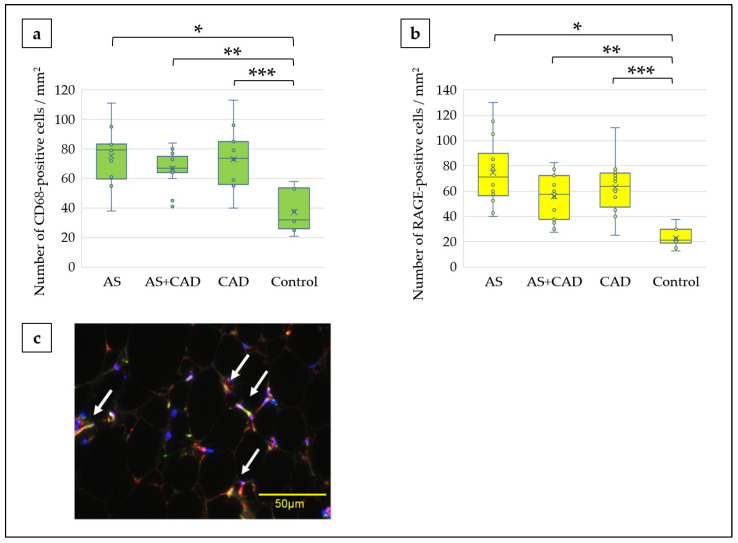
Quantification and colocalization of CD68- and RAGE-positive cells in EAT. (**a**) Number of CD68-positive cells per mm^2^. * AS vs. control: *p* < 0.001, ** AS+CAD vs. control: *p* < 0.001, *** CAD vs. control: *p* < 0.001. (**b**) Number of RAGE-positive cells per mm^2^. * AS vs. control: *p* < 0.001, ** AS+CAD vs. control: *p* = 0.001, *** CAD vs. control: *p* < 0.001 (**c**) Representative immunofluorescence images showing CD68 (green) and RAGE (red) colocalization; arrows indicate colocalized cells. Nuclei were stained with TO-PRO3 (blue). Scale bars: 50 µm. EAT, epicardial adipose tissue; AS, aortic stenosis; CAD, coronary artery disease; RAGE, receptor for advanced glycation end products.

Immunohistochemical analysis revealed that CD68- and RAGE-positive cells were localized around adipocyte nuclei in all surgical groups, while their expression was minimal in the controls (Figure 3a,b). Both the CD68-positive cells (each surgical group vs. control: *p* < 0.001) and RAGE-positive cells (AS vs. control: *p* < 0.001, AS+CAD vs. control: *p* = 0.001, CAD vs. control: *p* < 0.001) were significantly more abundant in the surgical groups than in the controls (Figure 4a,b). Immunofluorescence staining demonstrated CD68 and RAGE-positive cell colocalization (Figure 4c). No significant differences in CD68-or RAGE-positive cell counts were observed between the surgical groups.

### 3.4. Correlation Between the Number of Inflammatory Cells and the Clinical Variables

**Figure 5:**
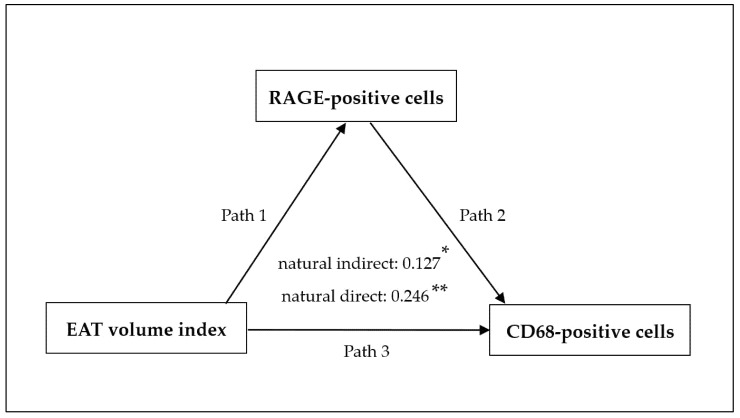
Directed acyclic graph of the causal mediation analysis. Causal mediation analysis demonstrating the relationship between the EAT volume index and CD68-positive cells. The model shows the natural indirect effect via RAGE-positive cells (paths 1 and 2) and the natural direct effect (path 3). Path estimates are presented in the center. * *p* = 0.024, ** *p* = 0.011. Path, pathway; EAT, epicardial adipose tissue; RAGE, receptor for advanced glycation end products.

Univariable analysis showed that the EAT volume index (β = 0.366, *p* < 0.001) and RAGE-positive cell counts (β = 0.407, *p* < 0.001) were significantly associated with CD68-positive cell counts. In the multivariable analysis (Table 2), both the EAT volume index (*p* = 0.018) and RAGE-positive cell counts (*p* = 0.011) remained significantly associated with CD68-positive cell counts in Model 1. However, after sequential adjustment for clinically relevant variables (Models 2–4), only the EAT volume index remained as the independent predictor of CD68-positive cell counts (*p* = 0.021). In causal mediation analysis, this index was associated with CD68-positive cells both directly and indirectly through RAGE-positive cells (Figure 5). Both the AS and AS+CAD groups demonstrated a correlation between C-reactive protein and the CD68- and RAGE-positive cell counts (Table 3). EAT volume index tended to be positively correlated with the triglyceride/HDL cholesterol ratio (*p* = 0.070). Notably, neither inflammatory cell counts nor the EAT volume index correlated with AS severity parameters (maximum aortic jet velocity, mean pressure gradient, or aortic valve area) (Table 3).

## 4. Discussion

This study revealed that the EAT volume index was significantly higher in the AS and CAD groups than in the controls, although it was similar across the surgical subgroups (Figure 2). Compared with the controls, the EAT from patients with AS, with or without concomitant CAD, exhibited increased macrophage infiltration and RAGE expression (Figure 3 and Figure 4). Notably, multivariable analysis identified that the EAT volume index was independently associated with CD68-positive cell counts (Table 2). In causal mediation analysis, a pathway was identified whereby the EAT volume index may be linked to CD68-positive cell activation via RAGE-positive cells (Figure 5); thus, AGEs–RAGE axis activation may contribute to local inflammatory reactions, as evaluated by macrophage infiltration within the EAT.

EAT volume positively correlates with aging and obesity-related parameters, including waist circumference and visceral fat [15,16]. Accumulating evidence suggests that increased EAT volume is strongly associated with atherosclerotic diseases, particularly in patients with diabetes mellitus and CAD [7,8]. Patients with CAD also exhibit significantly greater EAT volume and thickness than those without CAD, and EAT enlargement is associated with atherosclerosis severity [7,17]. Thus, EAT is not merely a passive marker of metabolic risk but may act as an active contributor to CAD disease progression, partly through inflammatory reactions that could further promote vascular injury [15]. Although AS is considered an atherosclerotic disease, its association with EAT remains unclear. In our study, the isolated AS and AS+CAD groups exhibited similarly increased EAT volume index, comparable to that in patients with CAD, and all surgical groups demonstrated significantly higher EAT volume than the controls. Furthermore, immunohistochemical analysis revealed that CD68- and RAGE-positive cell counts were significantly higher in the surgical groups than in the controls. Therefore, EAT may be involved in AS pathophysiology and disease progression through mechanisms similar to those observed in CAD.

Our findings extend previous observations linking EAT to CAD [8,9,18]. In CAD, EAT acts as an endocrine and paracrine organ that could release proinflammatory mediators, such as TNF-α, interleukin-6, and monocyte chemoattractant protein-1, into adjacent coronary vessels, thereby promoting atherogenesis [7,19]. Macrophage infiltration within the EAT could play a central role in this process. Therefore, increased EAT volume may correlate with CAD severity [7,15]. Conversely, the contribution of EAT to AS pathophysiology remains poorly understood. Parisi et al. reported that EAT from patients with calcified AS exhibits a proinflammatory phenotype, suggesting that metabolic stress contributes to valvular disease progression [19]. In our study, EAT in patients with AS exhibited enhanced macrophage infiltration as evaluated by CD68-positive cells and RAGE expression. This finding supports a potential mechanistic link between metabolic stress, local inflammation, and valvular pathology.

Importantly, CD68- and RAGE-positive cell colocalization (Figure 4c), together with our causal mediation analysis, suggests a potential pathway through which the AGEs–RAGE axis may be involved in macrophage infiltration within the EAT. This observation is consistent with the hypothesis that increased EAT volume may represent more than a marker of metabolic burden and may contribute to local inflammatory reactions, relevant to AS pathogenesis. This mechanism aligns with prior studies on vascular and metabolic disorders, in which activation of the AGEs-RAGE axis is associated with oxidative stress and inflammatory responses [20,21,22]. In this context, although the triglyceride/HDL cholesterol ratio did not differ significantly among the study groups, a trend toward a positive correlation was observed between the EAT volume index and the triglyceride/HDL cholesterol ratio (*p* = 0.070). Given that the triglyceride/HDL cholesterol ratio is considered a surrogate marker of insulin resistance-related dyslipidemia [23], this finding suggests a potential link between increased EAT volume and a metabolically vulnerable tissue milieu. Our results indicate that EAT may contribute to local valvular inflammation, rather than simply reflecting systemic metabolic derangements, thereby providing new insights into AS pathophysiology. Notably, inflammatory cell counts did not correlate with AS severity parameters (maximum aortic jet velocity, mean pressure gradient, or aortic valve area; Table 3); thus, EAT inflammation may precede or act independently of the hemodynamic progression of valvular stenosis.

Clinically, EAT volume and inflammatory activity may serve as complementary biomarkers for subclinical inflammation in AS. RAGE-positive cell and EAT volume measurements could complement conventional imaging and laboratory markers, potentially allowing the identification of patients at higher risk for inflammation-driven disease progression. Furthermore, therapeutic strategies targeting the AGEs-RAGE pathway could modulate local EAT inflammation, although this approach requires validation through prospective studies.

This study has several limitations. First, the sample size was relatively small, and all patients were recruited from a single center, possibly limiting the generalizability of the findings. Second, the cross-sectional design prevents the causal inference regarding the relationship between EAT inflammation and AS progression. Third, EAT samples were obtained from surgically accessible regions, which may not fully represent the entire epicardial fat depot. Conducting longitudinal studies with larger, multicenter cohorts is warranted to validate these findings and assess their clinical relevance.

## 5. Conclusions

In patients with AS, EAT exhibits enhanced macrophage infiltration and RAGE expression, making the AGEs-RAGE axis a potential contributor to local inflammatory reactions within the EAT. These findings provide new insights into potential mechanisms underlying the interplay between metabolic stress, EAT inflammation, and AS pathophysiology, highlighting EAT as a potential therapeutic target and biomarker in valvular heart disease.

## Figures and Tables

**Table 1:** Baseline characteristics of study participants.

	Isolated AS (*n* = 15)	AS+CAD (*n* = 15)	CAD (*n* = 12)	Control (*n* = 10)	*p* Value
**Patient’s Characteristics**					
Age, year	70.1 ± 15.1	74.7 ± 5.8	63.8 ± 11.2	62.8 ± 11.5	0.035
Female (%)	11 (73.3)	8 (53.3)	3 (12.0)	3 (30.0)	0.049
Body surface area	1.49 ± 0.20	1.54 ± 0.19	1.71 ± 0.22	1.59 ± 0.20	0.050
Heart rate, /min	66.4 ± 10.5	73.9 ± 15.5	65.0 ± 9.8	87.3 ± 13.5	<0.001
Systolic blood pressure, mmHg	128.7 ± 16.1	132.3 ± 19.3	122.1 ± 13.4	115.8 ± 16.6	0.088
Diastolic blood pressure, mmHg	75.1 ± 2.8	75.1 ± 2.8	69.0 ± 3.1	68.8 ± 3.4	0.251
Hypertension (%)	9 (60.0)	10 (66.7)	9 (75.0)	1 (10.0)	0.011
Dyslipidemia (%)	7 (46.7)	8 (53.3)	9 (75.0)	0 (0)	0.005
Diabetes mellitus (%)	2 (13.3)	5 (33.3)	8 (66.7)	2 (20.0)	0.023
Current smoking (%)	3 (20.0)	4 (26.7)	5 (41.7)	4 (40.0)	0.572
Hemodialysis (%)	1 (6.7)	2 (13.3)	0 (0)	1 (10.0)	0.622
Statins (%)	6 (40.0)	8 (53.3)	11 (91.7)	1 (10.0)	0.002
Calcium channel blockers (%)	7 (46.7)	5 (33.3)	4 (33.3)	0 (0)	0.098
ACEi/ARBs (%)	9 (60.0)	8 (53.3)	9 (75.0)	1 (10.0)	0.019
β-blockers (%)	5 (33.3)	7 (46.7)	8 (66.7)	1 (10.0)	0.049
**Blood test**					
White blood cell count, 1000/μL	4.9 ± 1.2	6.0 ± 1.6	6.5 ± 1.4	4.4 ± 2.7	0.017
Hemoglobin, g/dL	11.8 ± 1.6	12.0 ± 1.8	13.9 ± 1.7	10.8 ± 2.6	0.003
Platelet, 10,000/μL	19.1 ± 5.1	20.3 ± 6.4	21.5 ± 5.3	13.6 ± 7.4	0.533
CRP, mg/dL	0.17 ± 0.19	0.07 ± 0.04	0.18 ± 0.25	0.07 ± 0.02	0.175
Total cholesterol, mg/dL	189.8 ± 32.1	180.7 ± 34.6	175.6 ± 49.5	146.2 ± 40.3	0.059
LDL cholesterol, mg/dL	103.8 ± 26.2	99.1 ± 27.4	106.4 ± 37.3	59.7 ± 30.7	0.111
HDL cholesterol, mg/dL	61.7 ± 16.6	60.4 ± 19.4	44.5 ± 13.2	51.1 ± 13.3	0.043
Triglycerides, mg/dL	127.9 ± 64.4	107.7 ± 47.3	138.6 ± 72.2	147.3 ± 72.6	0.434
Triglyceride/HDL cholesterol ratio	2.3 ± 1.4	2.1 ± 1.6	3.3 ± 2.0	2.8 ± 2.0	0.293
Hemoglobin A1c, %	5.70 ± 0.40	6.10 ± 0.90	7.10 ± 1.10	5.50 ± 0.40	<0.001
Creatinine, mg/dL	1.3 ± 2.1	1.5 ± 1.7	0.8 ± 0.3	1.3 ± 1.2	0.739
eGFR, mL/min/1.73 m^2^	69.8 ± 33.7	59.6 ± 27.3	75.8 ± 21.7	62.3 ± 35.5	0.512
**Transthoracic echocardiography**					
Left ventricular diastolic diameter, mm	44.0 ± 8.2	46.7 ± 8.8	48.4 ± 8.9	42.6 ± 5.2	0.337
Left ventricular systolic diameter, mm	26.3 ± 5.6	33.0 ± 8.7	33.0 ± 9.9	26.0 ± 2.8	0.026
Left atrium diameter, mm	40.8 ± 8.2	38.0 ± 5.7	39.3 ± 4.8	30.3 ± 2.0	0.001
Interventricular septal thickness, mm	16.7 ± 14.7	12.1 ± 2.5	10.7 ± 1.6	9.2 ± 1.0	0.125
Posterior wall thickness, mm	13.3 ± 2.6	11.7 ± 1.8	9.1 ± 1.2	8.9 ± 1.1	<0.001
Left ventricular ejection fraction, %	66.4 ± 16.2	57.9 ± 12.1	59.5 ± 13.8	68.5 ± 8.1	0.154
Vmax, m/s	4.9 ± 1.0	3.8 ± 0.9			0.009
Mean pressure gradient, mmHg	53.4 ± 20.9	36.7 ± 17.4			0.033
Aortic valve area, cm^2^	0.63 ± 0.11	0.80 ± 0.21			0.012

Data are presented as mean ± SD, median (interquartile range), or *n* (%). Comparisons among groups were performed using ANOVA as appropriate. AS, aortic stenosis; CAD, coronary artery disease; ACEi, angiotensin-converting enzyme inhibitor; ARB, angiotensin receptor blocker; CRP, C-reactive protein; LDL, low-density lipoprotein; HDL, high-density lipoprotein; eGFR, estimated glomerular filtration rate; Vmax, maximum aortic jet velocity.

**Table 2:** Correlation between the number of CD68-positive cells in EAT and the clinical variables.

	Estimate	95% CI	*p* Value
<Model 1>			
EAT volume index	0.242	0.043, 0.443	0.018
RAGE-positive cells	0.274	0.065, 0.484	0.011
<Model 2> *			
EAT volume index	0.292	0.049, 0.536	0.020
RAGE-positive cells	0.218	−0.011, 0.447	0.062
<Model 3> **			
EAT volume index	0.304	0.050, 0.558	0.020
RAGE-positive cells	0.191	−0.055, 0.437	0.124
<Model 4> ***			
EAT volume index	0.307	0.050, 0.564	0.021
RAGE-positive cells	0.179	−0.090, 0.447	0.185

* Adjusted for Model 1, age, body surface area, and sex. ** Adjusted for Model 2, diabetes mellitus, dyslipidemia, and hemodialysis. *** Adjusted for Model 3 and CRP. 95% CI, 95% confidence interval; EAT, epicardial adipose tissue; RAGE, receptor for advanced glycation end products; CRP, C-reactive protein.

**Table 3:** Correlations between inflammatory cell counts in EAT and clinical variables in patients with AS and AS+CAD.

**<CD68-positive cells>**					
		Univariate			
	Category	Estimate	95% CI		*p* value
Sex	M	Ref			
	F	1.512	−4.686	7.710	0.621
Age		−0.052	−0.582	0.478	0.841
Body mass index		−0.009	−1.137	1.135	0.999
Hemodialysis		−7.885	−17.304	1.535	0.097
Statins		3.117	−2.832	9.066	0.292
ACEi/ARBs		3.053	−2.901	9.007	0.302
β blockers		3.428	−2.649	9.506	0.257
CRP		58.710	24.350	95.064	0.002
Total cholesterol		−0.130	−0.321	0.061	0.174
Triglycerides/HDL cholesterol ratio		2.360	−1.551	6.270	0.226
Hemoglobin A1c		−4.850	−14.467	4.772	0.309
eGFR		−0.065	−0.261	0.131	0.502
Number of RAGE-positive cells		0.020	−0.234	0.274	0.872
EAT volume index		−0.022	−0.298	0.253	0.869
Vmax		3.300	−2.225	8.826	0.229
Mean pressure gradient		0.153	−0.139	0.445	0.291
Aortic valve area		0.210	52.17	12.066	0.905
Postoperative atrial fibrillation		0.037	−0.018	0.106	0.192
**<RAGE-positive cells>**					
		Univariate			
	Category	Estimate	95% CI		*p* value
Sex	M	Ref			
	F	−4.211	−13.751	5.329	0.373
Age		−0.767	−1.534	0.0002	0.050
Body mass index		0.892	−0.840	2.623	0.300
Hemodialysis		6.506	−8.707	21.720	0.388
Statins		−4.706	−13.970	4.558	0.307
ACEi/ARBs		1.046	−8.394	10.485	0.822
β blockers		0.000	−9.684	9.684	1.000
CRP		64.793	6.845	122.741	0.030
Total cholesterol		−0.077	−0.402	0.246	0.629
Triglycerides/HDL cholesterol ratio		−0.408	−6.658	5.842	0.895
Hemoglobin A1c		−4.373	−10.927	10.181	0.542
eGFR		0.095	−0.210	0.400	0.528
Number of CD68-positive cells		0.049	−0.565	0.662	0.872
EAT volume index		0.113	−0.314	0.539	0.592
Vmax		3.042	−6.336	12.420	0.907
Mean pressure gradient		0.168	−0.322	0.658	0.953
Aortic valve area		−6.113	−60.985	48.760	0.820
Postoperative atrial fibrillation		0.014	−0.020	0.050	0.413
**<EAT volume index>**					
		Univariate			
	Category	Estimate	95% CI		*p* value
Sex	M	Ref			
	F	2.462	−6.142	11.066	0.563
Age		0.431	−0.288	1.149	0.230
Bodu surface area		20.792	−67.635	17.544	0.238
Hemodialysis		−8.884	−22.357	4.588	0.188
Statins		1.220	−7.128	9.569	0.767
ACEi/ARBs		2.669	−5.685	11.023	0.518
β blockers		2.884	−5.558	11.325	0.490
CRP		−3.820	−63.803	56.162	0.897
Total cholesterol		−0.034	−0.290	0.222	0.786
Triglycerides/HDL cholesterol ratio		4.887	−0.431	10.205	0.070
Hemoglobin A1c		−5.952	−19.510	7.606	0.375
eGFR		−0.024	−0.302	0.253	0.859
Number of CD68-positive cells		−0.046	−0.611	0.519	0.834
Number of RAGE-positive cells		0.096	−0.266	0.457	0.461
Vmax		−3.352	−10.663	3.960	0.354
Mean pressure gradient		−0.170	−0.596	0.255	0.417
Aortic valve area		−0.458	−49.277	48.362	0.985
Postoperative atrial fibrillation		0.039	−0.003	0.091	0.067

Ref, reference; ACEi, angiotensin-converting enzyme inhibitor; ARB, angiotensin receptor blocker; CRP, C-reactive protein; HDL, high-density lipoprotein; eGFR, estimated glomerular filtration rate; RAGE, receptor for advanced glycation end products; Vmax, maximum aortic jet velocity; EAT, epicardial adipose tissue.

## Data Availability

The original contributions presented in this study are included in the article. Further inquiries can be directed to the corresponding author.

## Abbreviations

The following abbreviations are used in this manuscript:

AGEs	advanced glycation end products
AS	aortic stenosis
CABG	coronary artery bypass grafting
CAD	coronary artery disease
CT	computed tomography
EAT	epicardial adipose tissue
RAGE	receptor of AGE
SAVR	surgical aortic valve replacement
TNF-α	tumor necrosis factor-α
